# Incorporating ecosystem services into functional zoning and adaptive management of natural protected areas as case study of the Shennongjia region in China

**DOI:** 10.1038/s41598-023-46182-0

**Published:** 2023-11-01

**Authors:** Zheng-yu Deng, Jia-shuo Cao

**Affiliations:** School of Ecology and Nature Conservation, Beijing Forestry University, Beijing, 100083 China

**Keywords:** Ecological modelling, Ecosystem ecology, Ecosystem services

## Abstract

Against the background of global climate change and anthropogenic interference, studying the spatial and temporal heterogeneity of ecosystem services in important ecological function regions and rationally dividing the functional zones will help to promote the construction of the natural protected areas system dominated by national parks. The Shennongjia Region is an important candidate for China's national parks and one of the key pilots. Integrating the InVEST model, Getis-Ord Gi* index, hotspot analysis, GeoDetector and K-means clustering algorithm, we measured five ecosystem services interactions and delineate the functional zones. The results show that the spatial and temporal evolution of various ecosystem services in the Shennongjia Region between 2000 and 2020 was significant. All ecosystem services showed a decreasing and then increasing trend, except for carbon storage, which slowly declined. The ecological status of the region is in the process of polarization, with the local environment showed a trend of continuous deterioration. Water yield-habitat quality and carbon storage-water purification showed synergistic relationships; soil conservation showed trade-offs with water yield, carbon storage and water purification over a wide spatial range. The interaction between land surface temperature and vegetation cover was the most significant dominant factor. Hot spots for the comprehensive ecosystem services index were mainly located in the central and southern parts of the Shennongjia region and four types of ecosystem service functional zones were identified accordingly. This study is of great significance for maximizing the benefits of ecosystem service functions, the efficient allocation of environmental resources and the rational formulation of management policies in natural protected areas.

## Introduction

Ecosystems provide various functions to humans, including provisioning, regulation, support, and cultural services. It is an important foundation for human survival and development and a safeguard for human well-being^1^. As economic and social development progress, the buffering and carrying capacities of natural ecosystems are being tested. According to the Millennium Ecosystem Assessment, 60% of ecosystems worldwide have been degraded by human activities^2^, resulting in the destruction of balanced land-use structures and affecting land efficiency and sustainable social development.

As people pay more attention to the ecological environment, the connotations and extensions of ecosystem services have deepened and become key research areas. Since Hughes^3^, Costanza^4^, and others began their research on ecosystem services, the research content, methods, models, and scales of this field have gradually improved. In terms of research content, the main focus has been on the quantitative valuation of ecosystem services^5^, functional monitoring^6^, relationships, and optimal management^7^. In terms of research methods, model analysis^8^, principal component analysis^9^, correlation analysis^10^, regression analysis^11^, energy value analysis^12^, scenario modelling^13^, and estimation of value equivalence tables^14^ have been applied. In terms of scales, it covers global^15^, national^16^, urban agglomeration^17,18^, river basin^19,20^, province^21,22^, city^23^, and region^24,25^. Ecosystem service research has become increasingly fruitful with the development of biophysical models and the application of high spatial resolution datasets^7^. In addition to static assessments, the spatial and temporal variability of ecosystem service values has received considerable attention. However, in the context of existing studies, the exploration of ecosystem services and their driving factors has been relatively simple, making it difficult to reveal the extent to which the factors behind the spatial variation in ecosystem service trade-offs are explained. Also, it is impossible to quantify the combination of driving factors due to complex geographical processes and evaluate their relationship with each other.

Ecosystem services have multiple characteristics, such as the diversity of service types, the non-equilibrium of service relations, and the variability of spatial distribution. However, owing to human socioeconomic activities and natural changes, different ecosystem services interact, and the relationships between ecosystem services change, with trade-offs or synergistic relationships emerging. Thus, how to effectively manage the trade-off and synergy between ecosystem services has become the focus of academic research^15^. Spatial mapping^26^, scenario analysis^27,28^, rose diagrams^29^, and model simulations^30^ are commonly used to investigate the spatial characteristics, scale effects, and impact mechanisms of trade-offs and synergies in ecosystem services. When exploring the relationship between ecosystem services, provisioning and regulating services are mutually suppressive in a trade-off relationship^24^. For example, good condition for vegetation growth can provide a high level of regulation services; however, the capacity for food production will be reduced, leading to a decrease in the level of provisioning services. Owing to regional variability in ecosystem service relationships, the trade-offs and synergies between different services vary in different study areas^31^. Thus, a proper understanding of the relationships between ecosystem services is a prerequisite for making decisions regarding the sustainable management of multiple ecosystem services, which contributes to the overall enhancement of human well-being. However, most methodological analyses are based on quantitative statistical relationships to reflect the overall regional variability, lacking the spatial expression of intra-regional differences, as well as research on the underlying mechanisms of ecosystem service relationship formation and the internal heterogeneity of natural ecosystems. Therefore, in this study, we explored the spatial and temporal pattern changes of ecosystem service trade-offs using a combination of the InVEST model, the Getis-Ord Gi* index, and hotspot analysis. We also explored the interactions and contributions of multiple driving factors using GeoDetector.

Ecological function zoning is the process of dividing an area into different ecological function zones according to its spatial heterogeneity on the basis of analyzing ecological conditions such as ecosystem characteristics and ecosystem service patterns, and its essence is ecosystem service regionalization. Bailey first proposed the concept of ecological zoning from the perspective of ecosystems^32^, and since then, scholars in various countries have strengthened the research related to ecological zoning, which has led to the rapid development of its theories and methods, and has been applied at the macro-regional scales^33^. Some of them have evaluated the ecological services by constructing the ecological importance index^34^ and the comprehensive index^35^ as the basis of functional zoning, while others have adopted the Self-Organizing Feature Map (SOFM) clustering analysis to classify the geographic data from its feature structure^36^, which has effectively avoided the problem of subjective judgment of zoning. At present, most scholars have carried out the delineation of ecological functional zoning at different scales, such as urban areas^37^ and river basins^38^, but there are few studies on the functional zoning of ecosystem services in natural protected areas with excellent ecological conditions. Ecological function zoning not only provides the necessary basis for the proper protection or utilization of the ecological environment, but is also an important scientific basis for guiding the development and management of natural protected areas.

Shennongjia is located in the transitional zone between northern and southern plant species in China and has the only well-preserved subtropical forest ecosystem and the richest biodiversity in the world's mid-latitudes; it is also one of the most important ecological functional areas and vulnerability zones worldwide^39^. However, due to rapid economic and societal development, especially tourism, the ecological environment of the Shennongjia Region has been strongly disturbed by human activities, showing large fluctuations at different spatial and temporal scales, leading to a series of ecological and environmental problems. In this study, the Shennongjia Region was selected as the object. Presently, studies on the ecosystems in the Shennongjia Region are mostly conducted on static, single-service types^40,41^, and few studies on dynamic, multiple ecosystem services, especially on the zoning of ecosystem service functions based on the results of multiple ecosystem service value assessments are available.

## Materials and methods

### Description of the study area

**Figure 1:**
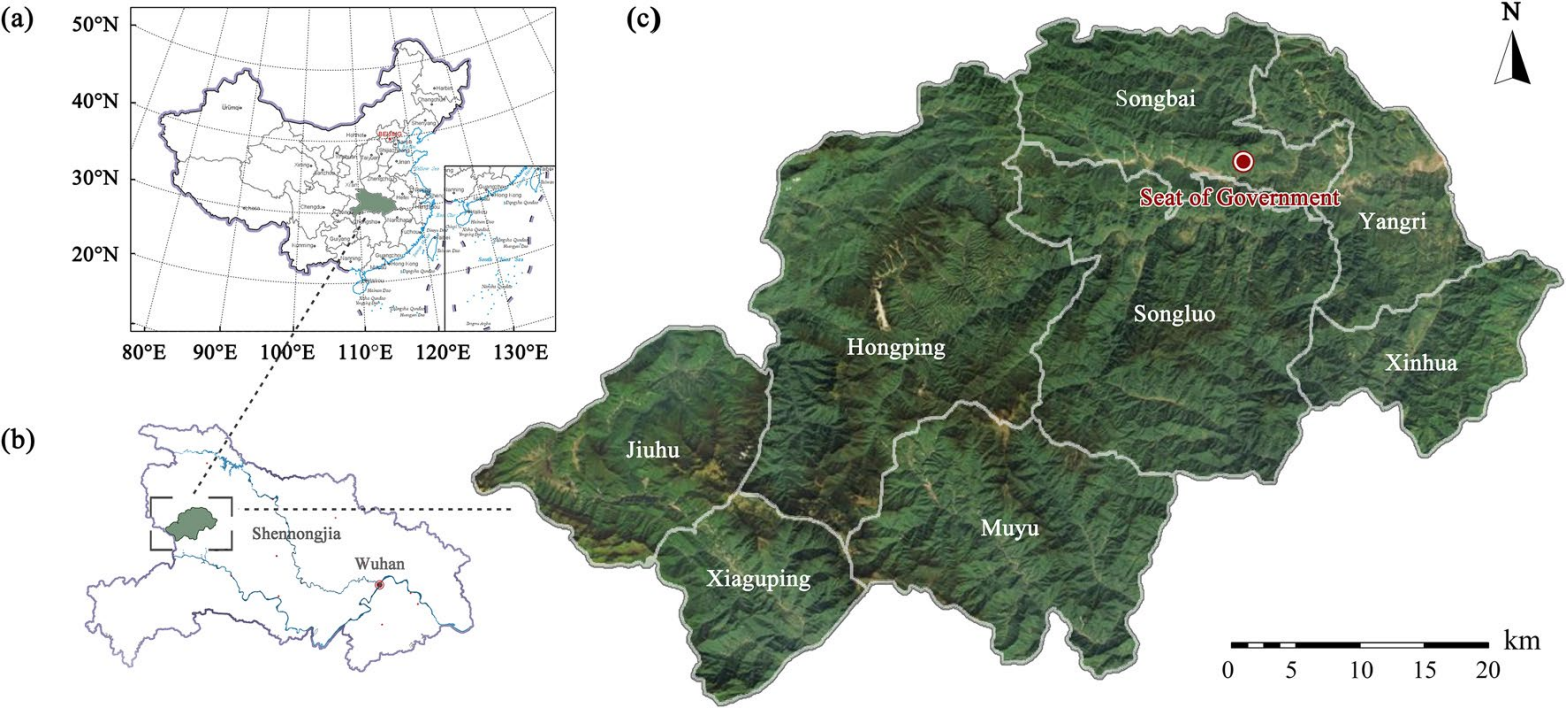
Location map of the Shennongjia Region. (**a**) Location of Hubei Province in China; (**b**) Location of Shennongjia Region in Hubei Province; and (**c**) Location of eight townships in the Shennongjia Region. The maps were generated by National Platform for Common Geospatial Information Services (https://www.tianditu.gov.cn/).

The Shennongjia Region is located in the northwestern mountainous region of Hubei Province, China (109°56′–110°58′ E, 31°15′–31°57′ N). It covers an area of approximately 3,215.80 km^2^ (Fig. 1). The southwestern part is dominated by east–west mountain ranges. Shennongjia has a subtropical monsoon climate characterised by distinct seasons, abundant precipitation, and evident vertical and horizontal climate zones. It receives an annual average of 1 858.3 h of sunshine, with precipitation (800–2500 mm per year) and evaporation (500–800 mm per year)^42^. Shennongjia preserves the most intact evergreen broadleaf mixed forests in the northern hemisphere. The ecosystem of these forests, composed of evergreen and deciduous broadleaf tree species, showcases the unique evolutionary processes of plant ecology. The Shennongjia forest area includes various ecosystems, such as forests, shrubs, meadows, and wetlands, providing important ecosystem services, such as climate regulation and water and soil conservation.

Owing to its uniqueness and representativeness in terms of biodiversity and ecological value, the Shennongjia Region is the first Chinese heritage site to be awarded the UNESCO World Natural Heritage, World Geopark, and Man and Biosphere Nature Reserve lists. Recently, China has vigorously promoted the construction and development of nature reserves, and the Shennongjia Region has been selected as one of the first pilot areas for the national park system, with the Shennongjia National Park soon to be among the next batch of national parks to be officially established.

### Analytical framework for ecosystem service interactions

**Figure 2:**
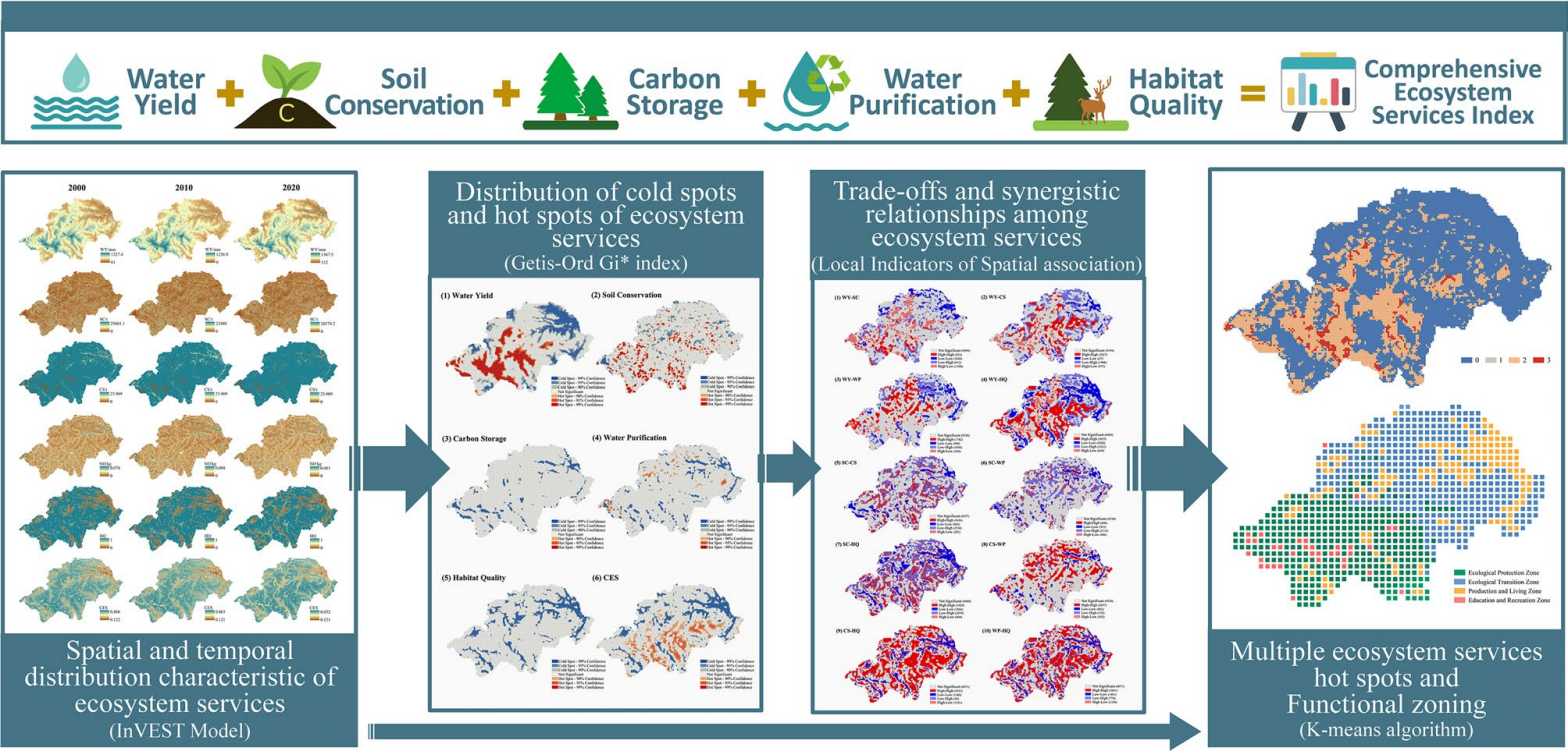
Analytical framework for ecosystem service interactions.

In this study, we developed a framework for quantifying the spatial and temporal heterogeneity of ecosystem service interactions. After data pre-processing, various ecosystem services were calculated using the InVEST model. We used the Getis-Ord Gi* index for hot and cold spots analysis and used GeoDA for Local Indicators of Spatial Association (LISA) analysis to identify trade-offs and synergistic relationships in ecosystem services. We probed for factors affecting ecosystem services with GeoDetector, and finally, the ecosystem service function zoning was carried out with K-means algorithm based on the above analysis. Detailed explanations of each step are provided in the subsequent sections (Fig. 2).

### Data sources

Eleven basic data points were used in this study: elevation, annual average precipitation, land use type, annual average reference evapotranspiration, available plant water content, maximum soil rhizome depth, rainfall erosivity factor, soil erodibility factor, land surface temperature, normalized difference vegetation index (NDVI) and population density (Table 1). Land use data were selected from the Landsat series satellite images for 2000, 2010, and 2020 and were obtained by interpretation. The overall interpretation accuracy reached 89.23%, which met this study requirement.

**Table 1:** Sources of basic data.

Data	Year	Type	Method and explanation	Sources
Elevation	2019	Raster	Digital Elevation Model (DEM)	Chinese Academy of Sciences Geospatial Data Cloud (http://wwwgscloud.cn)
Annual average precipitation	2000,2010,2020	Raster	1-km monthly precipitation dataset for China (1901–2021)	The Tibetan Plateau Scientific Data Center of the Chinese Academy of Sciences (https://data.tpdc.ac.cn/)
Land use types	2000,2010,2020	Raster	Six categories: Forest land; Grassland; Cultivated land; Construction land; Water area and Unutilized land	Website of USGS (http://glovis.usgs.gov/)
Annual average reference evapotranspiration	2000,2010,2020	Raster	Hargreaves Model	The Tibetan Plateau Scientific Data Center of the Chinese Academy of Sciences (https://data.tpdc.ac.cn/)
Available plant water content	2009	Raster	Harmonized World Soil Database (version 1.1) AWC_CLAS	Harmonized World Soil Database (HWSD) (https://www.fao.org)
Maximum soil rhizome depth	2009	Raster	Harmonized World Soil Database (version 1.1) REF_DEPTH	Harmonized World Soil Database (HWSD) (https://www.fao.org)
Rainfall erosivity factor	2000,2010,2020	Raster	Obtained from annual average precipitation	Chinese Academy of Sciences Resource and Environment Science and Data Center (http://www.resdc.cn)
Soil erodibility factor	2000,2010,2020	Raster	Obtained from the data of soil types and soil monitoring, using Erosion—productivity impact calculator (EPIC) Model	Chinese Academy of Sciences Resource and Environment Science and Data Center (http://www.resdc.cn)
Land surface temperature	2000,2010,2020	Raster	Daily 1-km all-weather land surface temperature dataset for the Chinese landmass and its surrounding areas (TRIMS LST; 2000–2021)	The Tibetan Plateau Scientific Data Center of the Chinese Academy of Sciences (https://data.tpdc.ac.cn/)
NDVI	2000,2010,2020	Raster	A 30-m annual maximum NDVI dataset in China from 2000 to 2020	National Ecosystem Science Data Center (http://www.nesdc.org.cn/)
Population density	2000,2010,2020	Raster	Population/land area	Shennongjia Bureau of Statistics

### Selection and assessment of ecosystem service

Based on the characteristics of the Shennongjia Region, five ecosystem services were selected and classified into three main categories for this study: provisioning services—water yield (WY); regulating services—carbon storage (CS), water purification (WP) and soil conservation (SC); and supporting services—habitat quality (HQ) (Table 2). We used ArcGIS10.6 software to quantify ecosystem services through the InVEST model^21,43,44^, and the description of the methods was given in Table 3.

**Table 2:** Types and selection basis of ecosystem services.

Category	Ecosystem services	Basis of selection
Provisioning services	Water yield (WY)	Both groundwater and surface water are influenced by the regulation of aquatic and terrestrial ecosystems and are essential for sustaining life on earth and for meeting the resource needs of the various ecological components within the ecosystem and for providing a continuous supply of water to the outside
Regulating services	Soil conservation (SC)	The Shennongjia Region is a transitional area for plant species from the north and south of China and a crossroads for many animals to flourish. It is an important ecological barrier in central China, possessing functions such as soil formation, water connotation and soil and water conservation
	Carbon storage (CS)	The carbon storage service is effective in mitigating the greenhouse effect and improving the living environment, which is of great significance in promoting the sustainable development of human society and mitigating global warming
	Water purification (WP)	Soil and vegetation, through physical and biochemical processes such as adsorption, transformation and post-washing, can play a purifying role in the pollutants entering the water environment, restoring some or all of the functions of the water body to its original state. The quality of water has a direct impact on human health and well-being
Supporting services	Habitat quality (HQ)	Habitat quality and scarcity not only reflect the level of biodiversity in a region, but also provide the species and genetic resources needed for ecological succession and biological evolution

**Table 3:** Description of the ecosystem services calculation methodology.

Ecosystem Services	Equations and description	
Water yield (WY) (m^3^)	$Y\left(x\right)=\left[1-\frac{AET(x)}{P(x)}\right]\times P(x)$	where *Y(x)* is the annual WY (m^3^) of grid cell *x*, *P(x)* is the annual precipitation (mm·a^-1^) of grid cell *x*, and *AET(x)* is the annual actual evapotranspiration of grid unit *x* (mm·a^-1^)
Soil conservation (SC) (t/hm^2^)	${SC}_{i}={RKLS}_{i}-{USLE}_{i}$ ${RKLS}_{i}={R}_{i}\times {K}_{i}\times {LS}_{i}$ ${USLE}_{i}={R}_{i}\times {K}_{i}\times {LS}_{i}\times {C}_{i}\times {P}_{i}$	where *SC*_*i*_ is the annual soil retention, *RKLS*_*i*_ is the annual potential soil erosion, *USLE*_*i*_ is the actual annual soil erosion, *R*_*i*_ is the rainfall erosivity factor, *K*_*i*_ is the soil erodibility factor, *LS*_*i*_ is the slope-slope length factor, *C*_*i*_ is the vegetation management factor, and *P*_*i*_ is the soil retention measurement factor
Carbon storage (CS) (t/hm^2^)	${C}_{t}={C}_{a}+{C}_{b}+{C}_{s}+{C}_{d}$	Where *C*_*t*_ is the total amount of carbon storage (*Mg C·ha*^*-1*^), *C*_*a*_, *C*_*b*_, *C*_*s*_ and *C*_*d*_ are the above-ground carbon storage (*Mg C·ha*^*-1*^), under-ground carbon storage (*Mg C·ha*^*-1*^), dead organic carbon storage (*Mg C·ha*^*-1*^) and soil organic carbon storage (*Mg C·ha*^*-1*^) in the study area, respectively
Nitrogen output (NO) (kg/hm^2^)	${ALV}_{x}={HSS}_{x}\times {plo}_{x}$ ${HSS}_{x}=\frac{{\lambda }_{x}}{{\lambda }_{w}}$	where *ALV*_*x*_ is the nitrogen output (NO) value, *HSS*_*x*_ is the hydrological sensitivity score, *plo*_*x*_ is the output coefficient, *λ*_*x*_ is the runoff index, and *λ*_*w*_ is the average runoff coefficient in the region. Generally, the higher the NO, the lower the WP capacity
Habitat quality (HQ)	${Q}_{j}={H}_{j}\left[1-\frac{{D}_{xj}^{z}}{{D}_{xj}^{z}+{k}^{z}}\right]$	where Q_xj_ is the HQ of grid unit *x* in land use type* j*, *H*_*j*_ is the habitat suitability of land use type *j*, *D*_*xj*_ is the habitat stress level of grid unit *x* in land use type *j*, *k* is a half-saturation factor, usually half of the maximum value of *D*_*xj*_, and *X* is constant, usually taking the value 2.5

The Comprehensive ecosystem services index (CES) was constructed as an indicator to quantify and compare the total supply of multiple ecosystem services (Wu et al., 2017). Based on previous experience and considering the actual situation in Shennongjia, the following weights were assigned to the ecosystem services: WY (0.25), SC (0.17), WP (0.12), CS (0.24), and HQ (0.22). The formula used is as follows:

1 $${CES}_{j}=\sum_{i=1}^{n}{w}_{i}\times {S}_{ij}$$

where ${CES}_{j}$ is the CES in year *j*, ${w}_{i}$ is the weight of the ecosystem service* i*, ${S}_{ij}$ is the normalised value in year *j*, and *n* is the number of types.

### Analysis of ecosystem services hot and cold spots

Hot spot analysis based on the Getis-Ord Gi* index can be used to identify spatial clusters of high (hot spots) and low (cold spots) values that are significant and to determine the location of spatial aggregations. The Gi* and *Z* values were calculated using the following formulas^45^:

2 $${G}_{i}^{*}=\frac{{\sum }_{j=1}^{n}{w}_{ij}{x}_{j}}{{\sum }_{j=1}^{n}{x}_{j}}$$

3 $$Z\left({G}_{i}^{*}\right)=\frac{{\sum }_{j=1}^{n}{w}_{ij}{x}_{j}-\overline{x}{\sum }_{j=1}^{n}{w}_{ij}{x}_{j}}{S\sqrt{\frac{\left[n{\sum }_{j=1}^{n}{w}_{ij}^{2}-{\left({\sum }_{j=1}^{n}{w}_{ij}\right)}^{2}\right]}{n-1}}}$$

4 $$S=\sqrt{\frac{\left[{\sum }_{j=1}^{n}{x}_{j}^{2}\right]}{n-1}}-{\left(\overline{x }\right)}^{2}$$

where* G* is the agglomeration index of patch *i*, *w*_*ij*_ is the weight matrix between patches *i* and *j*; *x*_*i*_ and *x*_*j*_ are the attribute values of patches *i* and *j,* respectively, *n* is the total number of patches, $\overline{x }$ is the mean value of all patches, and *S* is the standard deviation of the attribute values of all patches.

### Analysis of ecosystem service trade-offs and synergistic relationships

Bivariate spatial autocorrelation analysis was performed using ArcGIS to create 2 km × 2 km grids. Local Indicators of Spatial Association (LISA) were used to visualise the local correlation of the study area. The formula is as follows^46,47^:

5 $$I=\frac{n{\sum }_{i=1}^{n}{\sum }_{j=1}^{n}{w}_{ij}\left({y}_{i}^{m}-{\overline{y} }_{m}\right)\left({y}_{j}^{z}-{\overline{y} }_{z}\right)}{({\sum }_{i=1}^{n}{\sum }_{j=1}^{n}{w}_{ij}){\sum }_{i=1}^{n}\left({y}_{i}^{m}-{\overline{y} }_{m}\right)\left({y}_{j}^{z}-{\overline{y} }_{z}\right)}$$

6 $$I_{ij} = Q_{i}^{m} \mathop \sum \limits_{j = 1}^{n} \left( {w_{ij} Q_{j}^{m} } \right){ };Q_{i}^{m} = \frac{{y_{i}^{m} - \overline{y}_{m} }}{{\sigma_{m} }};Q_{j}^{z} = \frac{{y_{j}^{z} - \overline{y}_{z} }}{{\sigma_{z} }}$$

where *I* is the global bivariate spatial autocorrelation index; *n* is the number of grid units; *w*_*ij*_ is the spatial weight; ${y}_{i}^{m}$ is the value of attribute *m* of grid unit *i*; ${y}_{j}^{z}$ is the value of attribute *z* of grid unit *j*; ${\overline{y} }_{m}$ is the mean value of attribute *m*; ${\overline{y} }_{z}$ is the mean value of attribute *z*; *I*_*ij*_ is the local bivariate spatial autocorrelation index; ${\sigma }_{m}$ is the variance of attribute *m*; ${\sigma }_{z}$ is the variance of attribute *z*. The range of values for *I* is [− 1,1], with *I* > 0 and approaching 1 indicating a more significant synergistic relationship between ecosystem services, I = 0 indicating no trade-off/synergistic relationship between ecosystem services, and I < 0 and approaching −  indicating a more significant trade-off relationship between ecosystem services.

GeoDA was used to calculate the clustering maps of* I*_*ij*_ values to LISA. The ‘high–high’ and ‘low–low’ clusters represent synergistic relationships between ecosystem services, and the ‘high–low’ and ‘low–high’ clusters represent trade-offs relationships between ecosystem services.

### Analysis of driving factors of ecosystem services

We applied GeoDetector to analyse the extent to which factor explained the heterogeneity of ecosystem services in the Shennongjia Region, using ecosystem services as the dependent variable and driving factors as the independent variables. The strength of the explanation of each ecosystem service *Y* by the driving factor *X* in 2020 was determined based on the *q*-statistic.

**Figure 3:**
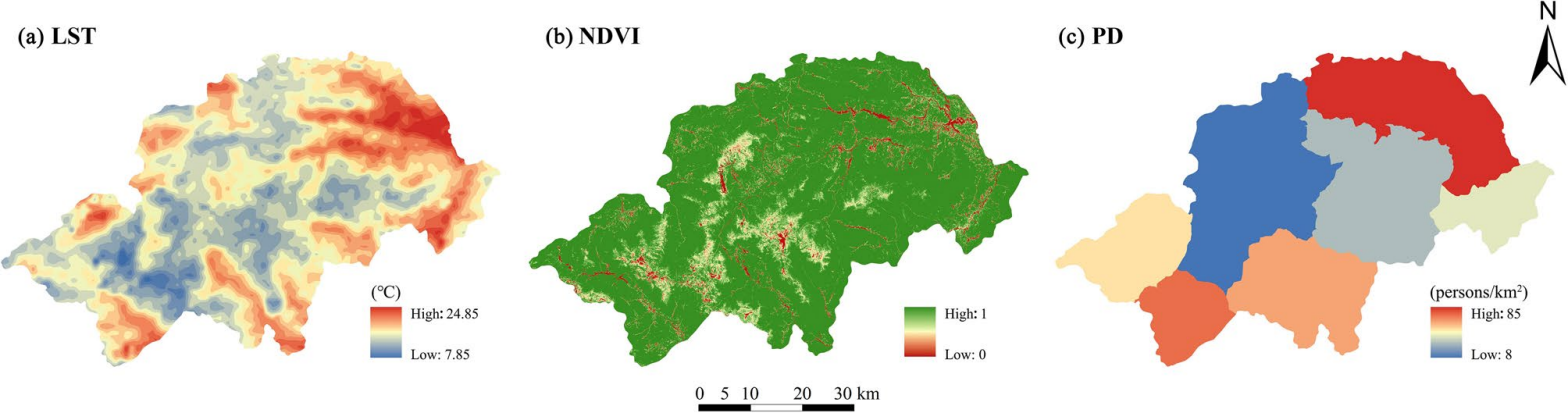
Key driving factors of ecosystem services in the Shennongjia Region. (**a**) LST: Land surface temperature; (**b**) NDVI: normalised vegetation index; (**c**) PD: Population density. The maps were generated by ArcGIS 10.6 (https://www.esri.com/en-us/arcgis/products/index).

Based on the analysis of the main factors related to each ecosystem service in the Shennongjia Region and data availability with reference to relevant research results^39,48^, land surface temperature, vegetation cover, and population density were selected as the key driving factors for factor detection and interaction detection (Fig. 3). The formula used is as follows^21^:

7 $$q=1-\frac{1}{N{\sigma }^{2}}{\sum }_{h=1}^{1}{N}_{h=1}{\sigma }_{h}^{2}$$

where *h* is the stratification of ecosystem service *Y* or driving factor *X*, *N*_*h*_ and *N* are the number of units in stratum *h* and the whole area, respectively, ${\sigma }_{h}^{2}$ and *σ*^2^ are the variance of stratum *h* and the whole area, respectively. *P*-values indicate the significance of the results, with smaller *p*-values indicating more significant results. *q* ranges from [0,1], with larger values indicating more significant spatial heterogeneity of ecosystem services.

### Functional zoning of ecosystem services

The ecosystem service bundle is a functional partitioning of ecosystem services obtained by measuring the similarity between different ecosystem services and classifying spatial units with high similarity into the same ecosystem service bundle and spatial units with high dissimilarity into different ecosystem service clusters. The main clustering methods for ecosystem service clusters are hierarchical clustering, K-means clustering, self-organising feature mapping network clustering, and random forest clustering^49^. Of these, K-means clustering is the most commonly used clustering method for continuous data because of its rapid data processing and concise and clear results. The K-means algorithm was used to achieve spatial clustering of ecosystem service clusters and functional zoning of the Shennongjia Region.

## Results

### Spatial and temporal evolution characteristics of ecosystem services of the Shennongjia Region between 2000 and 2020

#### Temporal characteristics of ecosystem services

Regarding time scale, the annual average values of WY services, SC, WP, HQ, and the CES of ecosystem services in the Shennongjia Region showed an overall decreasing and then increasing trend between 2000 and 2020, whereas CS showed a slow decreasing trend. Overall, the fluctuations in ecosystem services in the Shennongjia Region were minimal in the past 20 years. The area with high values increased, and the capacity for ecosystem services in the Shennongjia region increased steadily (Table 4).

**Table 4:** The annual average value of ecosystem services in Shennongjia from 2000 to 2020.

Ecosystem services	2000	2010	2020	Change between 2000 and 2020
Water yield (mm)	533.695	403.469	553.579	19.884
Soil conservation (t/hm^2^)	11,170.867	9220.156	11,298.581	127.714
Carbon storage (t/hm^2^)	261.574	260.850	259.834	− 1.740
Nitrogen output (kg/hm^2^)	0.080	0.081	0.076	− 0.004
Habitat quality	0.826	0.807	0.809	− 0.017
CES	0.630	0.615	0.621	− 0.009

CES: Comprehensive ecosystem services index.

#### Spatial characteristics of ecosystem services

Regarding spatial scale, the high-value areas for WY services in the Shennongjia Region were mainly located in the central and southern parts of Jiuhu, Xiaguaping, and Muyu townships. In the northeast, the WY value was generally low, while the town center of Songbai township produced a clear divergence from the surrounding area and the most pronounced increase in WY during the study period was identified. The high value areas for SC services were more sporadically distributed, whereas the low value areas were generally located in areas with low surface relief, which are mostly built-up township areas, cultivated areas, or areas where roads have been constructed. The high value areas for CS were widely distributed throughout the study area, whereas the low value areas were mainly located in the southwestern, central, and northeastern parts of the area, where the surface was more exposed and vegetation was sparse. The high values of NO were concentrated in areas with extensive cultivated land and high anthropogenic activity in the townships. The extent of the high values of NO decreased significantly between 2000 and 2020, indicating an improvement in the WP capacity of the Shennongjia Region.

**Figure 4:**
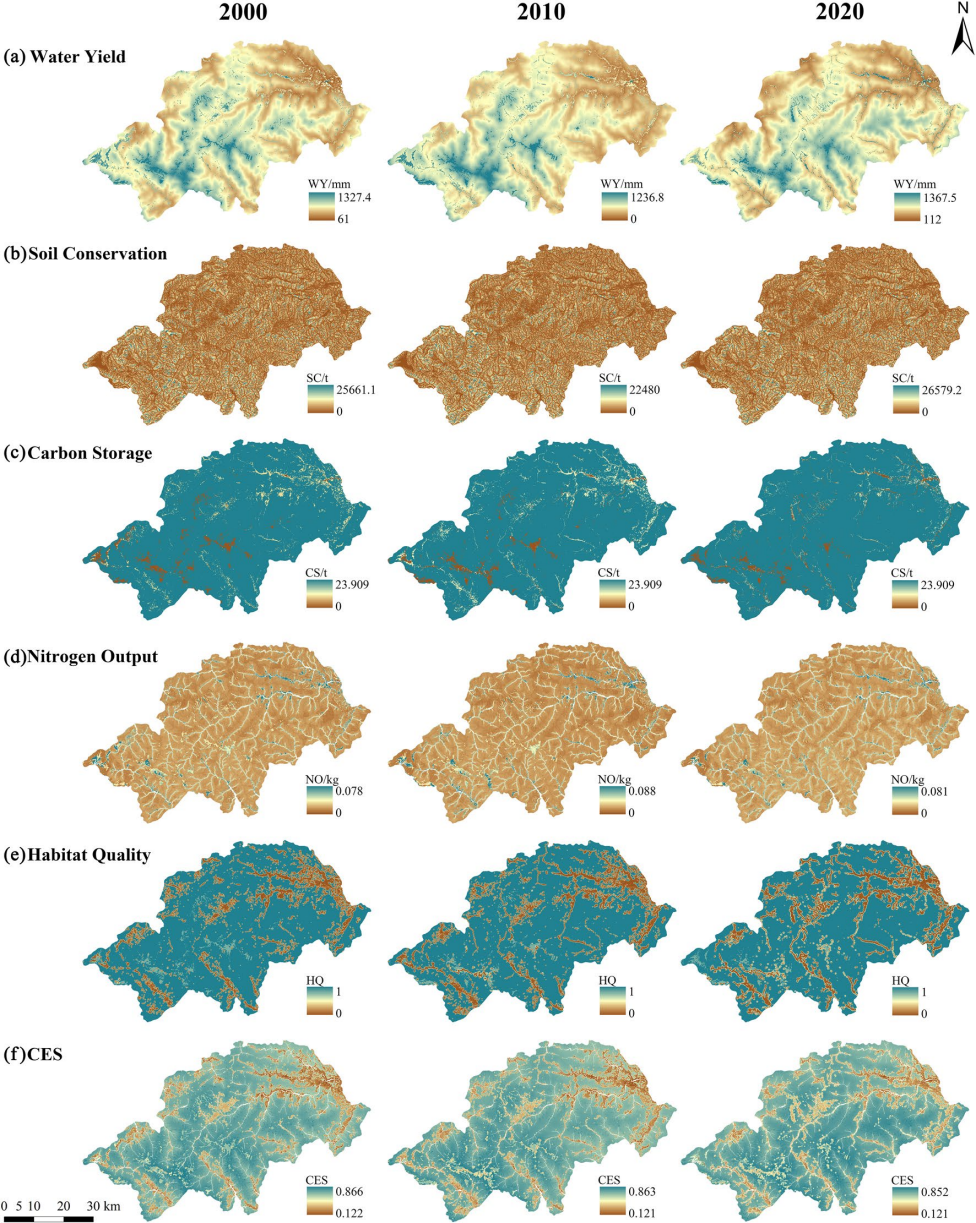
Spatial and temporal distribution and changes of ecosystem services in the Shennongjia Region from 2000 to 2020. CES: Comprehensive ecosystem services index. The maps were generated by InVEST 3.13.0 (https://naturalcapitalproject.stanford.edu/software/invest).

The spatial characteristics of HQ and the CES were similar in the overall distribution of low and high values in the northeast and southwest, respectively. The low value areas of HQ were concentrated in the main urban areas of each township and along the roads. In contrast, the high value areas were located in areas with higher vegetation cover and lower anthropogenic activities. Further, according to the spatial variation of the CES during 2000–2020, the ecological condition of the Shennongjia Region was in a polarisation process. On the one hand, the range of the low value areas of the CES was reducing, whereas the range of the high value areas was expanding, and the comprehensive capacity of ecosystem services and the ecological environment was improving. On the other hand, the CES in some of the low value areas significantly decreased; for example, the deterioration of the ecological environment was observed in local areas such as Muyu and Hongping townships (Fig. 4).

### Spatial distribution characteristics of cold and hot spots for ecosystem services in the Shennongjia Region

**Figure 5:**
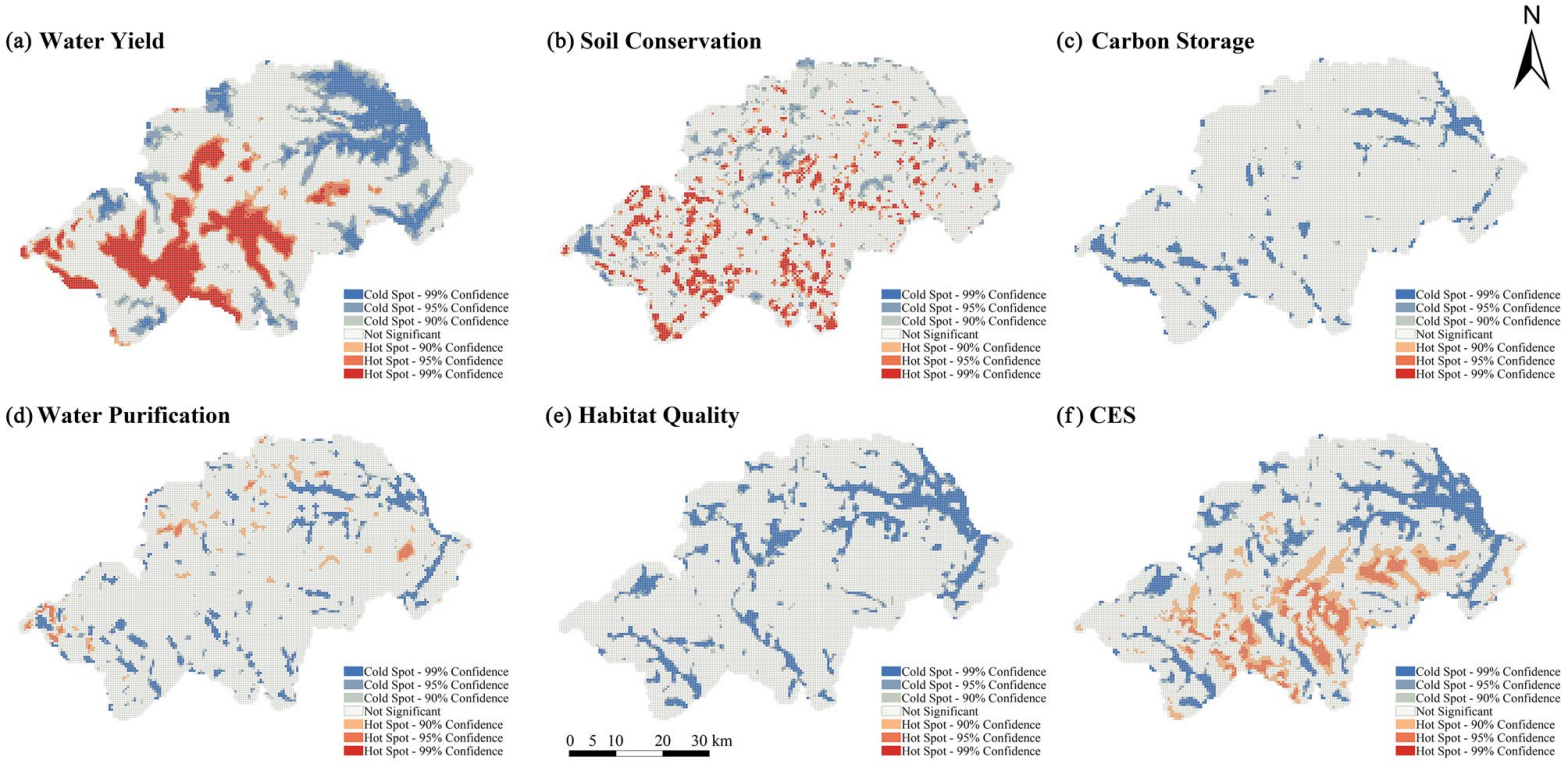
Distribution of cold spots and hot spots of ecosystem services in the Shennongjia Region. CES: Comprehensive ecosystem services index. The maps were generated by ArcGIS 10.6 (https://www.esri.com/en-us/arcgis/products/index).

Using the Gi* hotspot analysis tool, the spatial distribution of cold and hot spots for ecosystem services was mapped based on annual average values between 2000 and 2020 for the five ecosystem services and a comprehensive index (Fig. 5). The Shennongjia Region was classified into seven categories: extremely significant hot spots (99% confidence), significant hot spots (95% confidence), general hot spots (90% confidence), extremely significant cold spots (99% confidence), significant cold spots (95% confidence), general cold spots (90% confidence), and No significant.

Among the five ecosystem services, no hot spots for CS and HQ were identified, and the distribution of cold and hot spots for each ecosystem service was relatively similar, except for the WP hot spots, which accounted for only 3.63% of the area ranging from 9 to 24%. The proportion of hot spot areas was ranked as WY > SC > WP > CS and HQ, indicating that the aggregation effect of WY and SC was more evident in high value areas (Table 5). The proportion of cold spot areas was ranked as: WY > HQ > WP > CS > SC, indicating that the aggregation effect of WY and HQ was more evident in low value areas.

**Table 5:** The proportion of the cold and hot spots of ecosystem services in Shennongjia Region.

Ecosystem services	Hot spots (99%, 95%, 90% Confidence)		Cold spots (99%, 95%, 90% Confidence)	
	Spatial distribution	Proportion (%)	Spatial distribution	Proportion (%)
water yield	South, Central, West	19.75	Northeast	23.43
Soil conservation	West, South	13.08	Scattered	9.71
Carbon storage	–	–	Northeast, Southwest	10.09
Water purification	North, West	3.63	Northeast, South	10.49
Habitat quality	–	–	Northeast, South	19.38
CES	South, Central	17.00	Northeast, South	19.32

CES: Comprehensive ecosystem services index.

**Figure 6:**
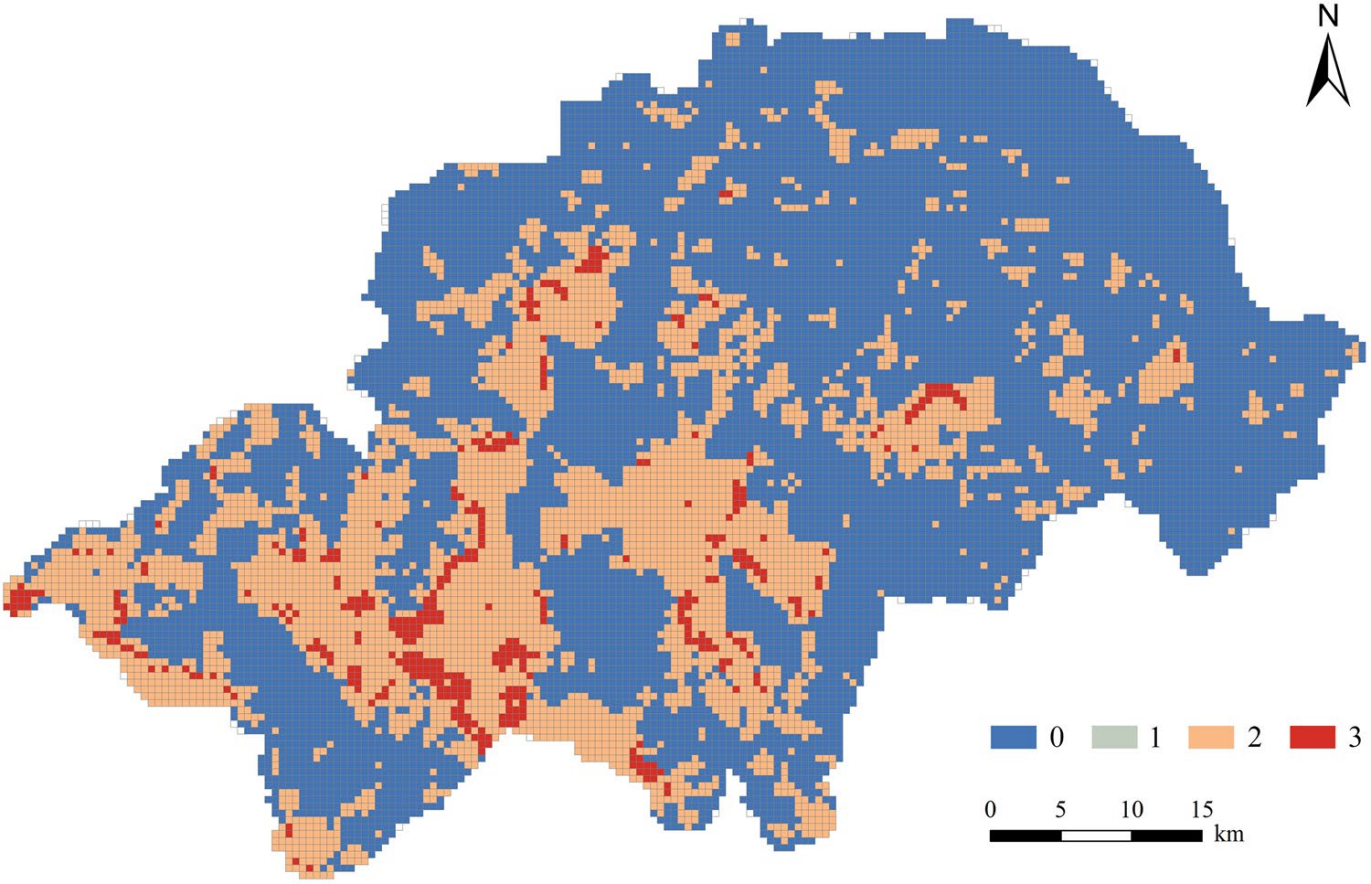
Spatial distribution of multiple ecosystem service hot spots in the Shennongjia Region. The maps were generated by ArcGIS 10.6 (https://www.esri.com/en-us/arcgis/products/index).

The spatial distribution of the various ecosystem services is characterised by variability, although a degree of overlap exists. The results of the overlay analysis of the hot spots of the five ecosystem services showed that at most three and at least zero ecosystem services overlapped in the same spatial extent in the Shennongjia Region (Fig. 6). The northeastern part of the Shennongjia Region had a concentration of cold spots for all ecosystem services except SC services. Hot spots for WY services were mainly located in the central, southern, and western parts of the region and were highly significant hotspots. These include the main urban areas of Muyu and Jiuhu townships and the Xiangxiyuan, Shennongding, and Nantianmen scenic areas. The cold and hot spots for SC services were widely distributed throughout the Shennongjia Region, whereas hot spots in the southern parts were more concentrated. No significant hotspots for CS and HQ were identified. The hotspots of the CES were mainly distributed in the central and southern parts of the region, and the cold spots were similar to that of HQ, concentrated in the northeast of Songbai, Yangri, and Xinhua townships and in the southern parts, where the main urban areas of Muyu and Xiaguping townships were located.

### Ecosystem service trade-offs and synergies of the Shennongjia Region

**Figure 7:**
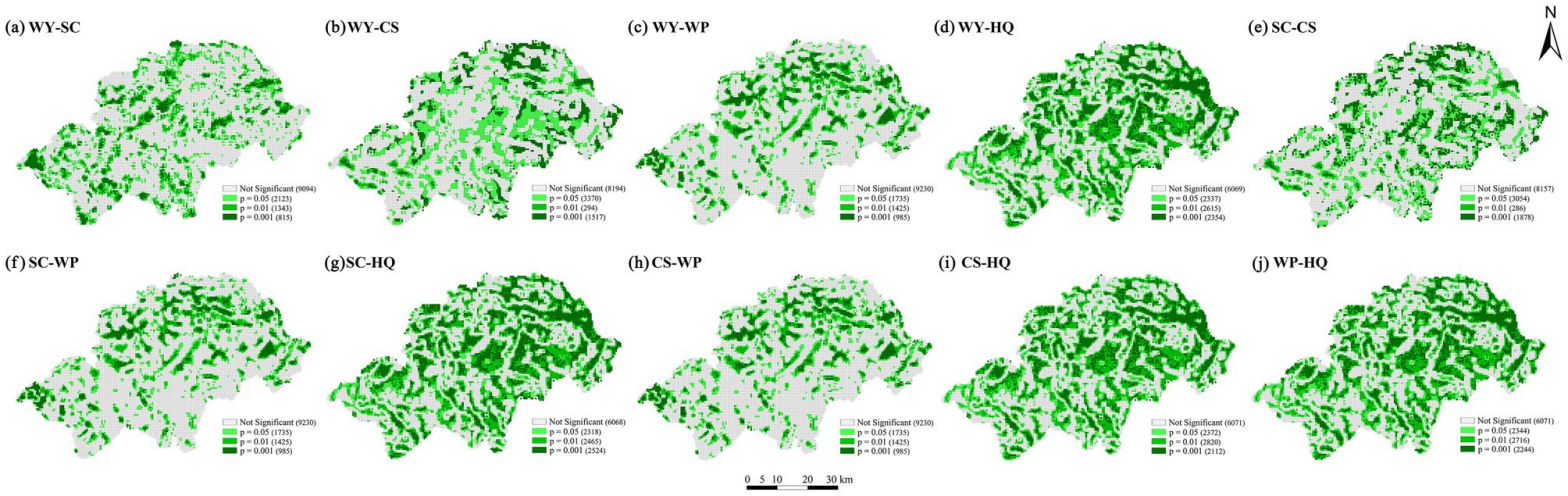
Bivariate local spatial autocorrelation significance of ecosystem services in Shennongjia Region. WY: Water yield; SC: Soil conservation; CS: Carbon storage; WP: Water purification; HQ: Habitat quality; CES: Comprehensive ecosystem services index. The maps were generated by GeoDa 1.22 (https://geodacenter.github.io/index-cn.html).

**Figure 8:**
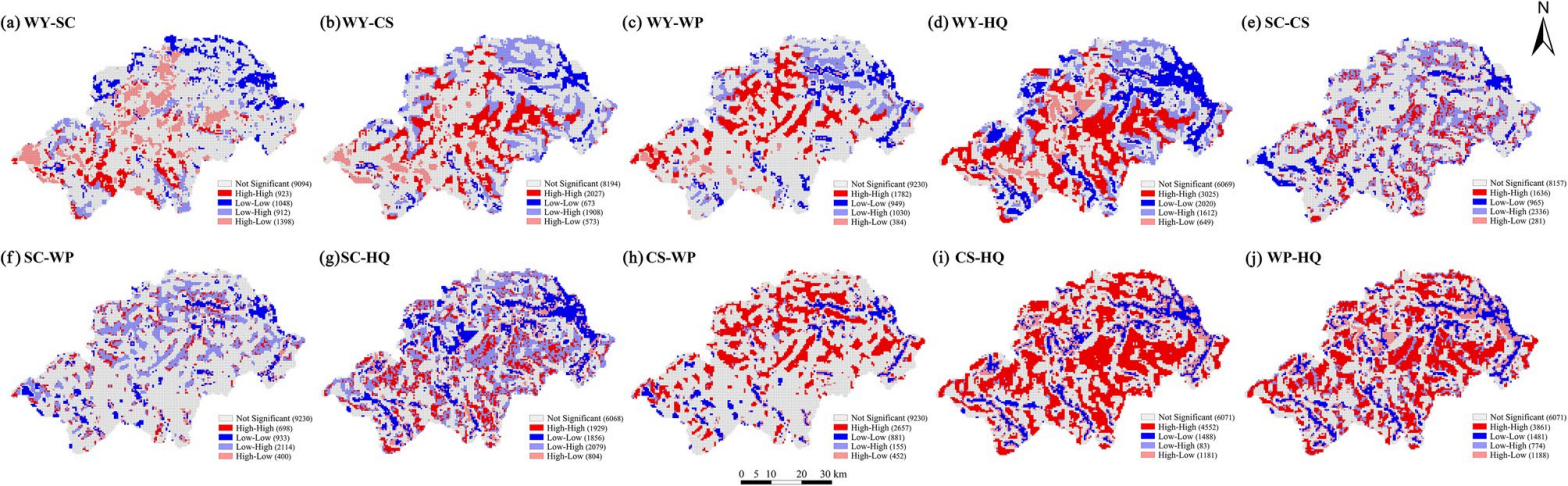
LISA cluster map between ecosystem services in Shennongjia Region. WY: Water yield; SC: Soil conservation; CS: Carbon storage; WP: Water purification; HQ: Habitat quality; CES: Comprehensive ecosystem services index. The maps were generated by GeoDa 1.22 (https://geodacenter.github.io/index-cn.html).

Any two ecosystem services were selected to calculate the bivariate local Moran'I index (Table 6). The ecosystem services in the Shennongjia Region exhibited different heterogeneities at different spatial scales (Fig. 7). The ‘high-high’ and ‘low-low’ values indicated that the two ecosystem services exhibited synergistic increasing and decreasing relationships in local areas, whereas the ‘low–high’ and ‘high-low’ values indicated that the two ecosystem services exhibited trade-offs relationships in local areas (Fig. 8).

**Table 6:** Moran’s I of local autocorrelation of ecosystem services in the Shennongjia Region.

Ecosystem services	SC	CS	WP	HQ
Water yield (WY)	− 0.022	− 0.028	0.102	0.218
Soil conservation (SC)	–	0.089	0.010	0.089
Carbon storage (CS)	–	–	0.253	0.283
Water purification (WP)	–	–	–	0.306
Habitat quality (HQ)	–	–	–	–

In the bivariate spatial autocorrelation analysis of ecosystem services in the Shennongjia Region, the distribution of ‘low-low’ values was highly similar, being located in the northeastern and parts of the southern regions where the values of ecosystem services were low and showed significant synergistic relationships with each other. WY and HQ, CS and WP, CS and HQ, and WP and HQ showed significant synergistic relationships in most of the regions. In the eastern parts of Hongping and Songluo townships and the northern part of Muyu Township, significant ‘high-high’ synergistic relationships were identified, whereas in the eastern parts of Songbai, Yangri, and Xinhua townships, significant ‘low-low’ synergistic relationships were discovered. The trade-off relationships between SC and WY and CS and WP occurred on a wide spatial scale. Of these, the trade-off relationship between WY and SC mainly occurred in the central region, that between SC and CS was widely distributed throughout the Shennongjia Region, and that between SC and WP mainly occurred in the northern region.

### Response relationship between ecosystem services and key driving factors of the Shennongjia Region

Three indicators not used for ecosystem service valuation were selected as key driving factors to analyze their impacts. As shown in Table 7, under the single factor detection, WY and HQ were most affected by land surface temperature, while SC, CS, and WP were most affected by NDVI. The values for each interaction factor were significantly higher than the single-factor detection results. They demonstrated both nonlinear and double-factor enhancements, indicating that multiple-factor interactions determine the degree of the trade-off relationship between ecosystem services. The interaction between land surface temperature and NDVI was the most significant interaction dominant factor for each ecosystem service. However, there is greater variability in the secondary key drivers affecting each ecosystem service. The interaction of land surface temperature and population density had a stronger effect on WP and HQ, while SC, CS, and WP were more strongly influenced by the interaction of NDVI and population density.

**Table 7:** Single factor detection and interaction detection of ecosystem services in the Shennongjia Region.

Ecosystem services	Factor detection		Interaction detection		Interact result
	Single factor	*q* value	Interaction factor	*q* value	
Water yield	x1	0.429	x1 ∩ x2	0.573	NE
	x2	0.111	x1 ∩ x3	0.547	DE
	x3	0.170	x2 ∩ x3	0.321	NE
Soil conservation	x1	0.016	x1 ∩ x2	0.100	NE
	x2	0.038	x1 ∩ x3	0.086	NE
	x3	0.033	x2 ∩ x3	0.093	NE
Carbon storage	x1	0.046	x1 ∩ x2	0.506	NE
	x2	0.319	x1 ∩ x3	0.122	NE
	x3	0.020	x2 ∩ x3	0.479	NE
Water purification	x1	0.041	x1 ∩ x2	0.283	NE
	x2	0.118	x1 ∩ x3	0.107	NE
	x3	0.014	x2 ∩ x3	0.211	NE
Habitat quality	x1	0.171	x1 ∩ x2	0.278	NE
	x2	0.075	x1 ∩ x3	0.206	NE
	x3	0.025	x2 ∩ x3	0.182	NE
CES	x1	0.318	x1 ∩ x2	0.409	NE
	x2	0.076	x1 ∩ x3	0.361	DE
	x3	0.072	x2 ∩ x3	0.215	NE

x1 = land surface temperature (LST); x2 = normalized difference vegetation index (NDVI); x3 = population density (PD); NE: Nonlinear enhancement; DE: Double factor enhancement; CES: Comprehensive ecosystem services index.

### Functional zoning of ecosystem services of the Shennongjia Region

The annual average values of the five ecosystem services and the CES were subjected to K-means cluster analysis. The inflection point effect of the sum of squares curves was more pronounced when the initial *k* value was 4; therefore, the Shennongjia Region was divided into four clusters, and the clustering results all passed the significance test. The annual average value of each ecosystem service was calculated for each of the four clusters (Table 8).

**Table 8:** Functional zoning of ecosystem service of the Shennongjia Region.

Cluster	Functional zones	WY	SC	CS	NO	HQ	CES
1	Ecological Protection Zone	588.871	1209.725	23.585	0.007	0.901	0.650
2	Ecological Transition Zone	500.649	887.672	23.785	0.006	0.892	0.638
3	Production and Living Zone	422.800	851.198	22.478	0.009	0.449	0.514
4	Education and Recreation Zone	760.835	657.795	20.945	0.012	0.743	0.614

WY: Water yield; SC: Soil conservation; CS: Carbon storage; NO: Nitrogen Output; HQ: Habitat quality; CES: Comprehensive ecosystem services index.

Cluster 1 had the highest SC, HQ, and CES values among the four clusters. CS and WY services were also high, with lower NO indicating higher WP services. Overall, this cluster had the highest value of ecosystem services, and combined with the distribution of hotspots, it was determined to have the highest ecological and conservation values. Therefore, it should be designated an Ecological Protection Zone.

Cluster 2 had the highest CS values among the four clusters; however, SC, WP, HQ, and CES values were second only to those of Cluster 1, indicating that this cluster also has a high ecological value. Considering that this area is in the transitional zone between Clusters 1 and 3 in terms of spatial distribution, it should be designated as an Ecological Transition Zone.

Cluster 3 had the lowest values for WY services, HQ, and the CES. Combined with the regional characteristics of its spatial distribution, it is the land on which cultivation and construction are mostly done. It is also the area with the highest intensity of anthropogenic disturbance and the longest development history in the Shennongjia Region. Therefore, it should be designated as a Production and Living Zone.

**Figure 9:**
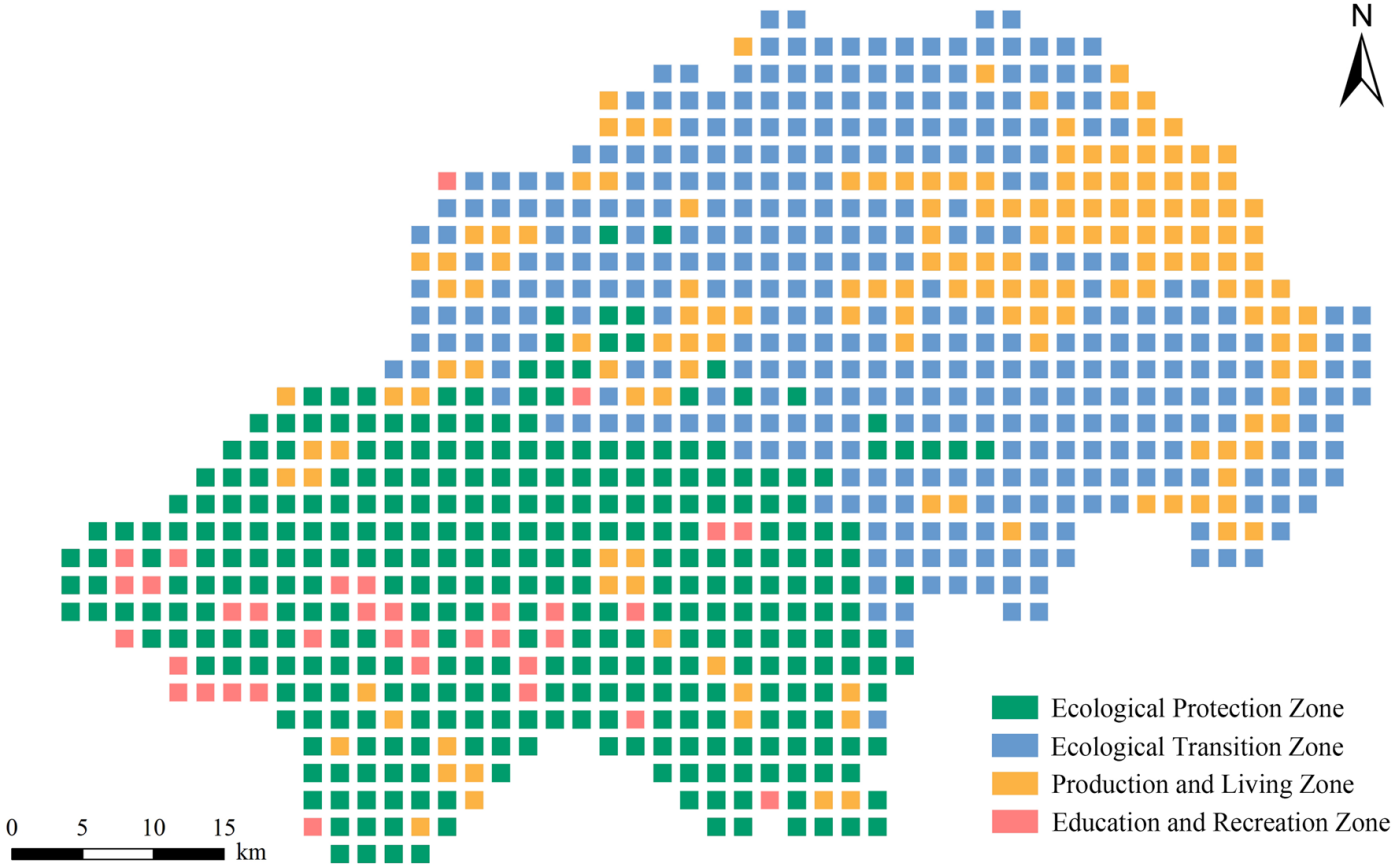
Functional zoning of ecosystem services in the Shennongjia Region. The maps were generated by Python (v3.10, https://www.python.org/).

Cluster 4 had the highest WY and NO values, and when combined with its spatial distribution, this cluster was scattered within the Ecological Protection Zone. In contrast to the current land use situation in the Shennongjia Region, this cluster is mostly a built-up area of the townships and is close to ecological tourism scenic spots, with beautiful natural scenery and good infrastructure; thus, it should be designated as the Education and Recreation Zone. Based on the results of this analysis, the functional zoning of ecosystem services in the Shennongjia Region was obtained (Fig. 9).

## Discussion

### Comparison of ecosystem services across land use types

Differences in land cover led to differences in ecological communities, which in turn resulted in different ecosystem service functions and ecological benefits. Although previous studies have revealed that the ecological vulnerability of forest land in the Shennongjia Region is mainly mild^39^, the health of forest ecosystems should be closely monitored, given their important ecological value. Forest land has a strong soil retention capacity, as it can intercept rainfall through the canopy, dead leaf, and soil layers, thus reducing the rainwater washout on the soil. The results of this study indicate that the increase in forest land after the implementation of the national ecological conservation projects has significantly improved CS, SC, and HQ.

WY is closely related to land use type. In the northeastern part of the Shennongjia Region, WY services are generally low, and there is only one high value area, Songbai township, where the Shennongjia Forestry District government is located, and Yangri township, which is adjacent to it. With urbanisation, there has been a continued expansion of construction land in the urban areas of Songbai and Yangri townships, with a significant shift from other land use types to construction land. The increase in impervious surfaces has greatly reduced the evapotranspiration of water bodies, resulting in a clear trend towards increased WY services in the area. The significant increase in WY may also increase the risk of flooding and soil erosion. At the same time, the high level of evapotranspiration from trees results in low WY from forest land.

The high value area for NO is located in the northern part of the Shennongjia Region, where the land use type is mainly cultivated land, probably because of the large amounts of chemical fertilisers used in agricultural production, which have high nitrogen and phosphorus contents. This also reflects the fact that cultivated land is the weakest in terms of WP compared to natural land cover types, such as forest land and grassland, which is consistent with the findings of existing studies^17^.

### Analysis of cold and hot spots in ecosystem services

The distribution of cold spots for WY services was somewhat similar to that of annual precipitation and topography, showing high and low distribution characteristics in the southwest and northeast, respectively. Precipitation was more abundant in the southwestern part of the Shennongjia Region due to the monsoon, and these areas are characterised by steep terrain and high mountain valleys, where precipitation can easily converge to form surface runoff^50^. The spatial distribution of cold spots for CS, WP, HQ, and the CES was concentrated in the northeast and south-central parts of the region, which have a long history of development and are subject to economic growth and anthropogenic activities. For example, Songbai township in the northeast is the seat of the Shennongjia Forestry District Government, whereas the Shennongjia Tourism Service Centre and the Shennongjia National Park Administration are located in Muyu township in the south-central region. This suggests that the uncontrolled expansion of urban land and rural settlements can cause a serious decline in ecosystem services and a significant reduction in HQ.

The overlay analysis revealed that the spatial distribution pattern of the comprehensive hotspot areas was highly consistent with that of forest land and grassland. For example, the areas in the south-central region, where forest land and grassland are mainly concentrated, are also significant hotspots for the CES, indicating that land use type is an important factor influencing the composite capacity of ecosystem services in the Shennongjia Region, consistent with the results of other related studies^7^.

### Analysis of trade-offs or synergies between ecosystem services

We observed significant synergistic relationships between HQ and WY and CS and WP services. The ‘high-high’ synergistic relationship between HQ and these three ecological services was mainly observed in the central part of the Shennongjia Region, an undulating mountainous area with low development intensity and anthropogenic activities. It also has high vegetation cover, and the soil consolidation effect of plant roots reduces soil erosion, resulting in high CS, WP, and HQ^51,52^. However, the high precipitation in this area, the relatively weak interception of surface runoff by vegetation on the hills, and the consequent increase in WY are consistent with the findings of previous studies^53^. The ‘low-low’ synergistic relationship was mainly discovered in these areas where the topography is flat, urbanisation is high, and the area of construction and cultivated lands is large, resulting in a combined decline in several ecosystem services, including HQ. Some studies have also shown that increased anthropogenic disturbances such as land expansion, tourism development, and grazing can cause a reduction in vegetation cover, CS services, and evapotranspiration from the land surface, and an increase in WY services^54,55^.

### Functional zoning of ecosystem services and control proposals

The Ecological Protection Zone is mainly located in the southern part of the Shennongjia Region, with a high capacity for various ecosystem services, high vegetation cover, and good ecosystem integrity, which are important for maintaining biodiversity. Notably, the scope of the Shennongjia National Park System Pilot Project highly overlaps with that of the Ecological Protection Zone delineated in this study. The Shennongjia National Nature Reserve and the Dajiu Lake Wetland Provincial Nature Reserve are also located within this functional zone, further confirming the high ecological value of the area. Therefore, the management objectives of this functional zone are focused on SC, climate regulation, and the maintenance of regional biodiversity. The central part of the Ecological Protection Zone, inlaid with the Production and Living Zone, is the main urban area of Muyu township, mainly carrying the ecological tourism industry of the Shennongjia Region and is more intensely disturbed by anthropogenic activities. In summary, activities such as agricultural production or infrastructure construction should be strictly controlled in the surrounding areas to avoid disorderly development and reduce damage to natural vegetation. The construction and management of national parks and other protected areas should be continuously promoted, and ecological compensation mechanisms should be actively implemented and improved. Simultaneously, efforts should be made to enhance the integrity and connectivity of the upgraded forest land patches within this functional zone to safeguard the proliferation and exchange of biological species within the region and maintain regional ecological security.

The Ecological Transition Zone is mainly located in the northern part of the Shennongjia Region, between the Ecological Protection Zone and Production and Living Zone, and plays a transitional and buffering role. This functional zone should be established on maintaining the stability of ecosystem services, focusing on restoring areas damaged by anthropogenic disturbances. The restoration of wildlife habitats, which are of national importance containing large areas of artificial vegetation, should be based on natural forces, supplemented by artificial interventions where necessary. Simultaneously, ecological corridors should be built to link isolated areas with important natural ecosystem distributions and enhance ecosystem connectivity.

The Production and Living Zone is mainly located in the northeastern part of the Shennongjia Region, with a scattered distribution in the central and southern parts. This functional zone is dominated by construction and cultivated lands. During urban expansion, it is important to protect the integrity of ecological land and repair the boundaries of existing construction land to prevent ecological disturbance and erosion. New construction spaces should be reasonably controlled, and the grouping of construction land should be promoted to achieve intensive development. In production activities, it is important to avoid crude agricultural production methods and focus on SC and water conservation while avoiding the misuse of pesticides and chemical fertilisers to reduce nitrogen and phosphorus loads in water bodies. Over the past 20 years, 70% of the increase in cultivated land in China resulted from the reclamation of rural settlements, which is a more scientific and effective approach than indefinitely increasing the area of cultivated land. Similarly, existing research suggests that improving the quality of the remaining cultivated land is more important than maintaining a quantitative balance^56^.

The Education and Recreation Zone is scattered throughout the southwestern part of the Shennongjia Region, with a high ecosystem service capacity; however, it is also subject to strong anthropogenic disturbances. This functional zone has high value for natural endowments and beautiful natural landscapes. It is also an area where ecotourism activities are performed more intensively than in the Shennongjia Region. For example, the highest peaks in Central China, Shennongding, and the scenic area where it is located, as well as the Dajiu Lake wetland, are in the Education and Recreation Zone. Therefore, without damaging or destroying natural resources, this functional zone can be appropriately used for scientific and educational recreational activities, such as scientific research and monitoring, natural environment education, ecotourism, and forest recreation.

### Implications and innovations

In the context of global climate change and human interference, the spatial and temporal evolution characteristics of ecosystem services have been studied, the trade-offs and synergistic relationships between ecosystem services are correctly perceived, and the driving factors are identified, aiding the scientific and effective regulation and management of various ecosystem services^57^. In this study, we integrated existing research to construct a complete and mature framework that can be used in ecosystem service evaluation and other related endeavours. We explored the spatial and temporal pattern changes of ecosystem service trade-offs using a combination of the InVEST model, the Getis-Ord Gi* index, and hotspot analysis and the interactions and contributions of multiple driving factors using GeoDetector.

Rational delineation of the functional areas of ecosystem service and differentiated management and control suggestions based on the dominant service types of each functional area are of great significance to maximize the benefits of ecosystem service functions, efficient allocation of environmental resources, and the rational formulation of ecological and environmental policies in natural protected areas. We proposed a quick method for functional zoning of ecosystem services with K-means clustering algorithm to identify ecosystem service clusters. This framework and method are based on the spatial and temporal heterogeneity and takes into account the intrinsic mechanisms of ecosystem service relationship formation, which have universal applicability to all kinds of natural protected areas including national parks, nature reserves and so on.

### Limitations and perspectives

Firstly, due to the limitation of data acquisition, we only selected five indicators: WY, SC, CS, WP and HQ, to characterize ecosystem services, which did not fully reflect the ecological values and functions of some regions. In the future, we can further enrich the types of ecosystem services by including indicators of food production, air purification and cultural services, and give new connotation to ecosystem services from the perspective of ‘socio-economic-natural ecosystems’, so that we can explore the underlying driving mechanisms in a quantitative manner, and provide scientific references for exploring the solution of sustainable key issues such as green economy, ecological protection, and public shared eco-tourism in important ecological function zones. Secondly, the diversity of landscape composition and landscape structure at different spatial scales makes the mutual mechanisms of ecosystem services heterogeneous. In the future, the analysis of the multi-scale response of ecosystem services to landscape patterns will be of great significance to the optimization of regional landscapes and the enhancement of ecosystem service capacity. It will be more conducive to helping managers make scientific decisions and achieve the enhancement of management capacity under the framework of regional sustainable development.

## Conclusion

Exploring the spatial and temporal variability of ecosystem services, and delineating the functional zones of ecosystem services accordingly, is an important basis for ecosystem management and regulation. Six key points were derived from this study: (1) The spatial and temporal evolution of various ecosystem services in the Shennongjia Region between 2000 and 2020 was significant. All ecosystem services showed a decreasing and then increasing trend, except for carbon storage, which slowly declined. (2) The ecological status of the region is in the process of polarisation. The local environment showed a trend of continuous deterioration. (3) Hot spots for the comprehensive ecosystem services index were mainly located in the central and southern parts of the region. (4) Water yield-habitat quality, carbon storage-water purification, carbon storage-habitat quality, and water purification-habitat quality demonstrated significant synergistic relationships majorly; soil conservation showed trade-offs with water yield, carbon storage, and water purification over a wide spatial range. (5) The interaction between land surface temperature and vegetation cover was the most significant dominant factor affecting each ecosystem service. (6) The Shennongjia region was divided into four types of ecosystem service functional zones, and recommendations for differentiated ecological control were proposed for each zone. The method for functional zoning of ecosystem services was proposed, which was based on the spatial and temporal heterogeneity of ecosystem services and takes into account the intrinsic mechanisms of ecosystem service relationship formation.

## Data Availability

The datasets used and/or analysed during the current study available from the corresponding author on reasonable request.
